# Functional Linkage of RKIP to the Epithelial to Mesenchymal Transition and Autophagy during the Development of Prostate Cancer

**DOI:** 10.3390/cancers10080273

**Published:** 2018-08-16

**Authors:** Mahmoud Ahmed, Trang Huyen Lai, Sahib Zada, Jin Seok Hwang, Trang Minh Pham, Miyong Yun, Deok Ryong Kim

**Affiliations:** 1 Department of Biochemistry and Convergence Medical Sciences and Institute of Health Sciences, Gyeongsang National University School of Medicine, Jinju 527-27, Korea; ma7moud_sha3ban@hotmail.com (M.A.); tranghuyen20493@gmail.com (T.H.L.); s.zada.qau@gmail.com (S.Z.); cloud8104@naver.com (J.S.H.); phamminhtrang010895@gmail.com (T.M.P.); 2 Department of Bioindustry and Bioresource Engineering, College of Life Sciences, Sejong University, Seoul 05006, Korea; myyun91@gmail.com

**Keywords:** RKIP, PEBP1, autophagy, EMT, cancer, microarrays, TCGA, WGCNA

## Abstract

Raf kinase inhibitor protein (RKIP) plays a critical role in many signaling pathways as a multi-functional adapter protein. In particular, the loss of RKIP’s function in certain types of cancer cells results in epithelial to mesenchymal transition (EMT) and the promotion of cancer metastasis. In addition, RKIP inhibits autophagy by modulating LC3-lipidation and mTORC1. How the RKIP-dependent inhibition of autophagy is linked to EMT and cancer progression is still under investigation. In this study, we investigated the ways by which RKIP interacts with key gene products in EMT and autophagy during the progression of prostate cancer. We first identified the gene products of interest using the corresponding gene ontology terms. The weighted-gene co-expression network analysis (WGCNA) was applied on a gene expression dataset from three groups of prostate tissues; benign prostate hyperplasia, primary and metastatic cancer. We found two modules of highly co-expressed genes, which were preserved in other independent datasets of prostate cancer tissues. RKIP showed potentially novel interactions with one EMT and seven autophagy gene products (TGFBR1; PIK3C3, PIK3CB, TBC1D25, TBC1D5, TOLLIP, WDR45 and WIPI1). In addition, we identified several upstream transcription modulators that could regulate the expression of these gene products. Finally, we verified some RKIP novel interactions by co-localization using the confocal microscopy analysis in a prostate cancer cell line. To summarize, RKIP interacts with EMT and autophagy as part of the same functional unit in developing prostate cancer.

## 1. Introduction

The loss of the Raf kinase inhibitor protein (RKIP), also known as a phosphatidylethanolamine binding protein 1 (PEBP1), is connected to the development of metastatic prostate cancers as well as a few other types of aggressive cancers [1]. The low expression of RKIP/PEBP1 in cancer cells results in transcriptional and post-transcriptional modifications of proteins such as SNAI1 and NF-κB and consequently promotes the process of the epithelial to mesenchymal transition (EMT), which is an early step of cancer metastasis [2]. Additionally, RKIP/PEBP1 is also associated with regulation of starvation-induced autophagy through the modulation of LC3-lipidation and mTORC1 signaling [3].

The key processes in cancer development can be explored by screening the gene–gene interactions and common regulators of their individual members. These potential interactions constitute a valuable resource for formulating hypothesis and informing further experimental works. High-throughput experimental datasets can be fed in an analysis pipeline such as the weighted-gene co-expression network analysis (WGCNA) to detect modules/networks of highly co-expressed genes [4]. The modules that correlate with the samples’ phenotypes signify the important and consequential networks of interacting genes [5]. Networks that are preserved across independent datasets are more likely to be biologically meaningful [6].

In this study, we identified two modules of highly co-expressed gene products involved in phosphatidylethanolamine binding (PEB), autophagy and EMT from a gene expression dataset of progressing prostate cancer. The term PEB is the molecular function that includes several gene products that take part in and regulate autophagy such as PEBP1/RKIP and MAP1LC3B/LC3. The two modules were preserved in several other independent datasets of prostate cancer. Moreover, we described novel interactions involving RKIP/PEBP1 and their contribution in autophagy and EMT processes. Finally, we found several upstream regulators of RKIP/PEBP1 and its binding partners in the context of prostate cancer.

## 2. Results

### 2.1. Expression Datasets and Gene Annotations

We identified a microarray dataset that consists of 13 cancer tissues of human prostate. The samples are classified by histological staining and expression signature into three distinct groups: benign prostate tumor, primary and metastatic cancer [7]. Three groups in this analysis reflect the alterations of gene expression in the prostate tumor as it progresses from the benign to the more aggressive forms. In addition, we used several independent gene expression RNA-Seq datasets of prostate cancer tissues as a validation set (Table 1). The validation datasets were obtained from the National Cancer Institute (NCI) genomic data center. We limited the analysis and validation to the 153 probes and the genomic regions that map uniquely to the members of three gene ontology (GO) terms: phosphatidylethanolamine binding (PEB), epithelial to mesenchymal transition (EMT) and autophagy (Supplementary Table S1). GO terms systematically define and index the gene products corresponding to their molecular functions and biological processes.

### 2.2. Module Detection of Interconnected Genes

**Figure 1:**
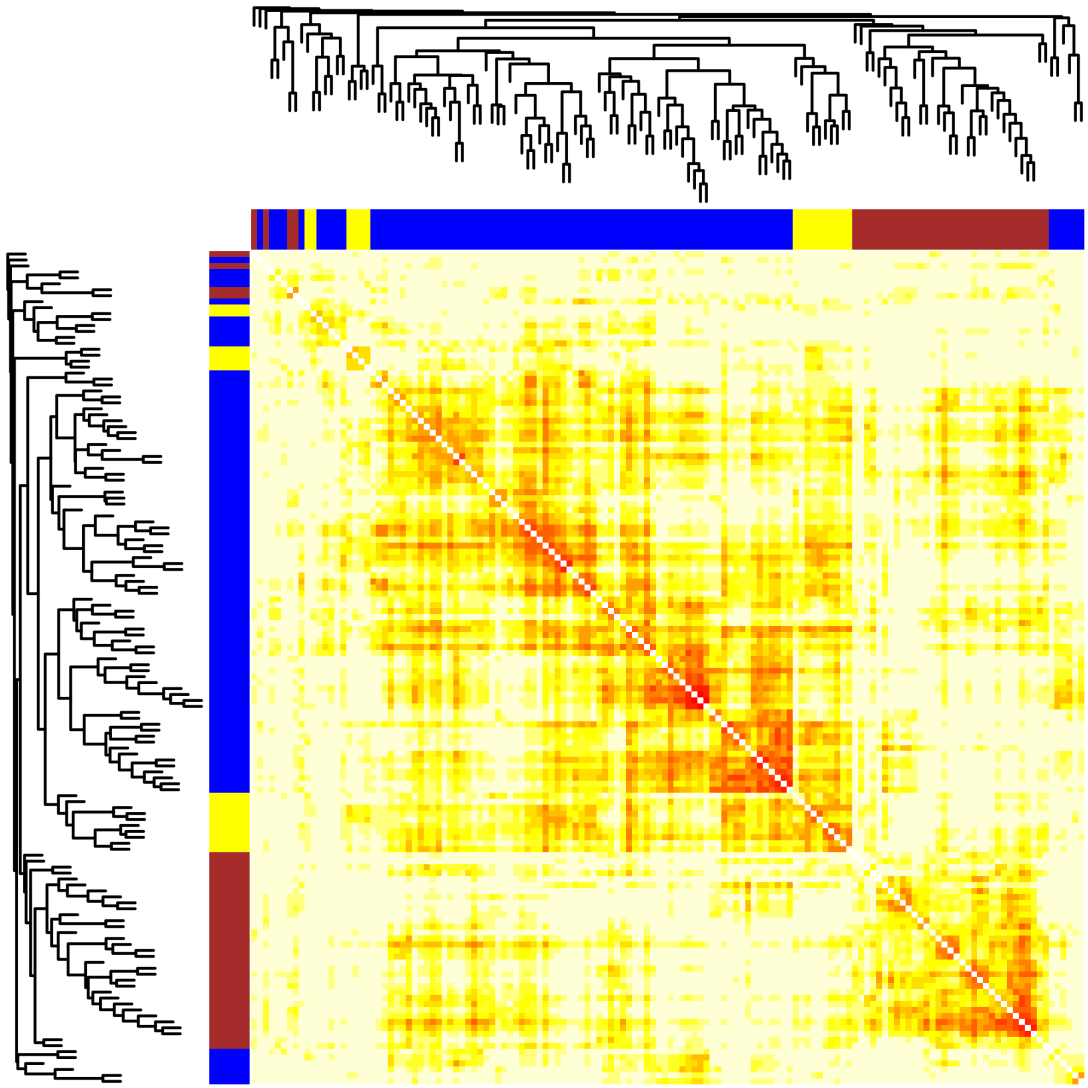
Clustering of epithelial to mesenchymal transition (EMT), phosphatidylethanolamine binding (PEB) and autophagy genes by their pairwise distances. Pairwise topological overlap matrix (TOM) similarities of PEB, EMT and autophagy genes (*n* = 142) were calculated from their expression values in the GSE3325 dataset. Distances between each pair of genes were derived as 1-TOM and shown as color values (small, *red* or large, *yellow*). A hierarchical tree and colored segments of the clusters were shown on the *top* and *side*.

In order to assess the co-expression of PEB gene products with the members of the EMT and autophagy gene sets, we applied WGCNA on the microarray dataset and found 142 gene products matched to at least one probe on the arrays. First, the expression values of 142 probes in 13 prostate tissue samples were used to calculate the pairwise Pearson’s correlation coefficients. The resulting 142 × 142 adjacency matrix was raised to the fifth power to discount very weak correlation values (Figure 1). Second, the discounted matrix was used to compute a similarity measure called the topological overlap matrix (TOM). Finally, the TOM similarity was used to derive two useful measures: weights and distances. The weight of an edge between a pair of genes is the strength of their connection in a network of all possible pairs. The distance (1-TOM) between two genes determines how likely they belong to the same functional network. Using average distance-based hierarchical clustering, an appropriate number of clusters/modules was determined and each gene was assigned to its closest (Supplementary Figure S1).

The 142 gene products were clustered in three distinct modules (blue, 87; brown, 37 and yellow, 18) based on their expression profiles in the different samples (Table 2). Members of the same modules were highly correlated with each other than with those of other modules. Therefore, the modules approximate independent functional sub-networks. Notably, gene products from different GO terms were distributed among modules. This indicates that the influence of one process on the other during the progression of prostate cancer might be mediated by more than a single pathway. Moreover, RKIP/PEBP1 was assigned to the brown module that contained EMT as well as autophagy genes, hence it is likely to interact with both as part of the same functional unit.

### 2.3. The Correlation of Modules with the Sample Phenotype

**Figure 2:**
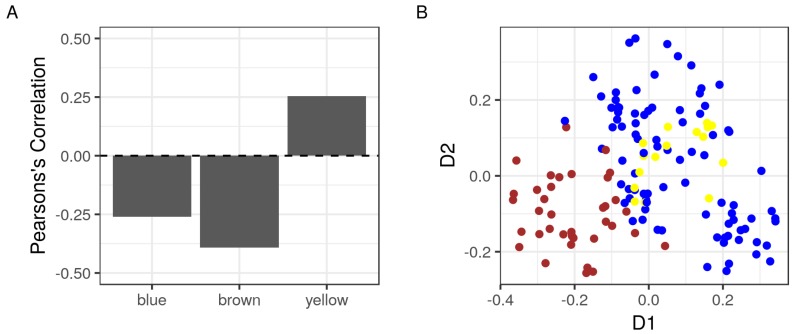
Correlations of detected modules to the sample phenotype and to each other. The expression values of the members of detected modules in the GSE3325 dataset (87, *blue*; 37, *brown*; and 18, *yellow*) were used to calculate the principal component (PC) for each module as a whole. (**A**) the Pearson’s correlations of the modules’ first PC and the phenotype of the samples of origin; and (**B**) the first (D1) and second (D2) PC of three modules shown as points. Colors represent the corresponding modules.

The biological significance of detected modules can be further illustrated by showing how well they correlate with the sample phenotype. To do that, we used the expression values of individual members of each module to calculate representative summaries, principal components (PC), for the modules as wholes. The first PC of three modules was correlated reasonably with the phenotype of the samples (>0.25 in absolute value) (Figure 2A). In addition, the modules were well separated along at least one of two dimensions (PC1 and PC2) (Figure 2B). Together, these three modules are likely to be significant and fairly independent functional units.

### 2.4. Module Preservation in Independent Datasets

**Figure 3:**
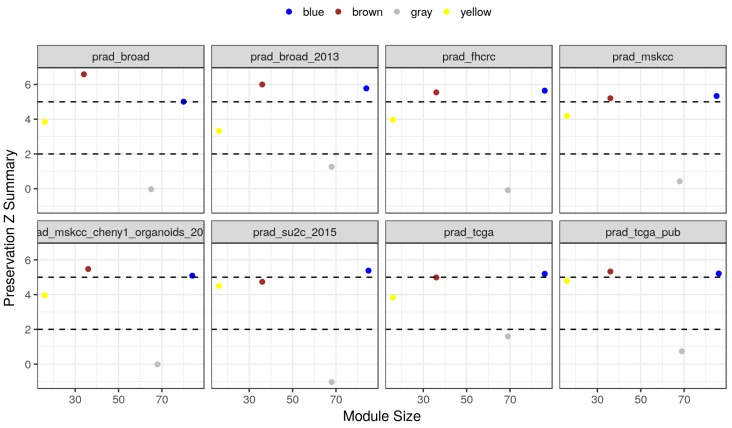
Module preservation Z summary across multiple prostate cancer datasets. The GSE3325 dataset was used to detect the highly co-expressed modules among PEB, EMT, and autophagy genes (87, *blue*; 37, *brown*; 18, *yellow*; and *gray*, randomly assigned). The detected modules were used as a reference to calculate several preservation statistics in eight independent datasets of prostate cancer. Z summary statistics and sizes of four modules are shown as colored points.

We tested the preservation of three modules in eight independent gene expression datasets of human prostate cancers (Table 1). The preservation test included several network statistics represented as a composite score called Z summary. Values between 5 and 10 are considered moderately preserved, while more than 10 are considered highly preserved. Two modules, brown and blue, were found to be moderately preserved in all eight datasets with a Z summary score of 5 or more (Figure 3). Modules in this category are generally reproducible and biologically meaningful. By contrast, the yellow and the gray (randomly assigned) modules showed much lower scores. In addition, the preservation ranks of modules relative to each other were shown in Supplementary Figure S2.

### 2.5. The Potential Interactions of RKIP/PEBP1 with Autophagy and EMT Gene Products

To further investigate the interactions of RKIP/PEBP1 with EMT and autophagy gene products, we first applied a minimum edge weight threshold (0.1) to the co-expression network. Then, we isolated the interactions involving one or more of the PEB gene set members (Supplementary Table S2). In particular, RKIP/PEBP1 showed potential interactions with seven autophagy and one EMT gene product (Table 3). The autophagy gene products included members of multiple key protein families (PIK3C3, PIK3CB, TBC1D25, TBC1D5, TOLLIP, WDR45, WIPI1), and the transforming growth factor beta receptor 1 (TGFBR1) was isolated as an EMT gene product. Additionally, we queried the STRING database for previously reported protein-protein interactions of RKIP/PEBP1. Text-mining analysis of published literature showed the connection of RKIP/PEBP1 with several proteins such as PARK7, CTNNB1 and NFI (Supplementary Table S3).

**Figure 4:**
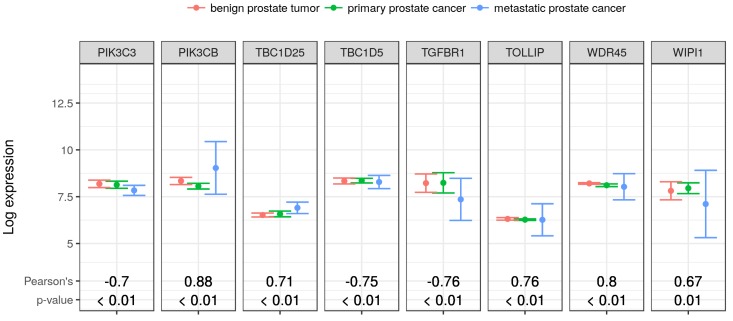
Expression profiles and correlations of gene products connected to RKIP/PEBP1 in developing prostate cancer. Eight gene products were identified to be potentially interacting with RKIP/PEBP1 during the progression of prostate cancer. The expression profiles (average ± SD) of eight genes in 13 samples (4, benign prostate tumor; 5, primary prostate cancer; and 4 metastatic prostate cancer) were shown. The Pearson’s correlation coefficients of eight gene products with RKIP/PEBP1 were calculated along with the *p*-values of correlation.

Although the TOM is a reliable similarity measure for identifying and clustering co-expressed genes, the Pearson’s correlation provides a straightforward measure for the magnitude and direction of the correlations between each pair of genes. In Figure 4, we showed the expression profiles and correlations of eight novel interacting partners with RKIP/PEBP1. Interestingly, two PI3K kinases PIK3C3 and PIK3CB showed a high expression correlation with RKIP/PEBP1, but they were in opposite directions (−0.7 and 0.88 of Pearson’s coefficient, respectively; *p* < 0.01). This expression correlation between RKIP/PEBP1 and PI3K families will be discussed later. WIPI1 showed a similar expression pattern to TGFBR1, and both were strongly correlated with RKIP/PEBP1 (Pearson’s coefficient, 0.67 and −0.76, respectively; *p* < 0.01). Although the expression of TBC1D25, TBC1D5, TOLLIP and WDR45 did not vary much across the sample groups, each had a strong correlation with RKIP/PEBP1 (absolute Pearson’s coefficient >0.7; *p* < 0.01).

### 2.6. Common Regulators of RKIP/PEBP1 and Its Interacting Partners

**Figure 5:**
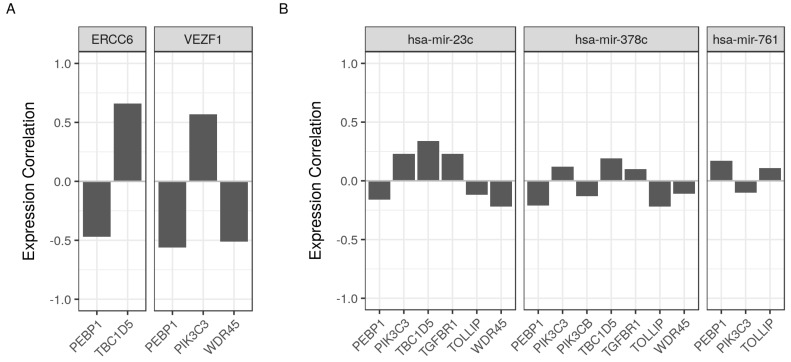
Common regulators of RKIP/PEBP1 and related gene products in prostate cancer. Regulatory factors (transcription factors and microRNAs) in prostate cancer were surveyed for the ones that correlate and/or bind to RKIP/PEBP1 and at least one of its eight related gene products. (**A**) Expression correlation of two transcription factors and their target genes. (**B**) Expression correlation of three microRNAs and RKIP/PEBP1 and correlated gene products.

Two highly co-expressed genes are likely to have biologically meaningful relation such as binding physically, being part of the same complex or having common upstream regulators. To explore the last possibility, we queried the cRegulome database to identify transcription factors and microRNAs that target the *RKIP/PEBP1* gene and one or more of EMT and autophagy genes in prostate cancer (preparing for publication). The targets of transcription factors were determined based on the integrative analysis of ChIP-seq and RNA-Seq data of human prostate cancer tissues. The microRNAs were correlated with coding genes using total RNA-Seq of cancerous prostate samples. From these analyses, we identified two transcription factors and three microRNAs that target the *RKIP/PEBP1* gene and at least one of eight genes (Table 4). Excision repair 6, chromatin remolding factor (ERCC6) suppressed the expression of *RKIP/PEBP1* gene, while it increased the expression of *TBC1D5* gene. Similarly, vascular endothelial zinc finger 1 (VEZF1) suppressed the *RKIP/PEPB1* and *WDR4* expression but had the opposite influence on the *PIK3C3* expression (Figure 5A). Three human microRNAs; miR-23c, miR-378c and miR-761, were correlated with the expression of five, six and two out of the eight genes of interest in addition to the expression of *RKIP/PEBP1* gene (Figure 5B).

**Figure 6:**
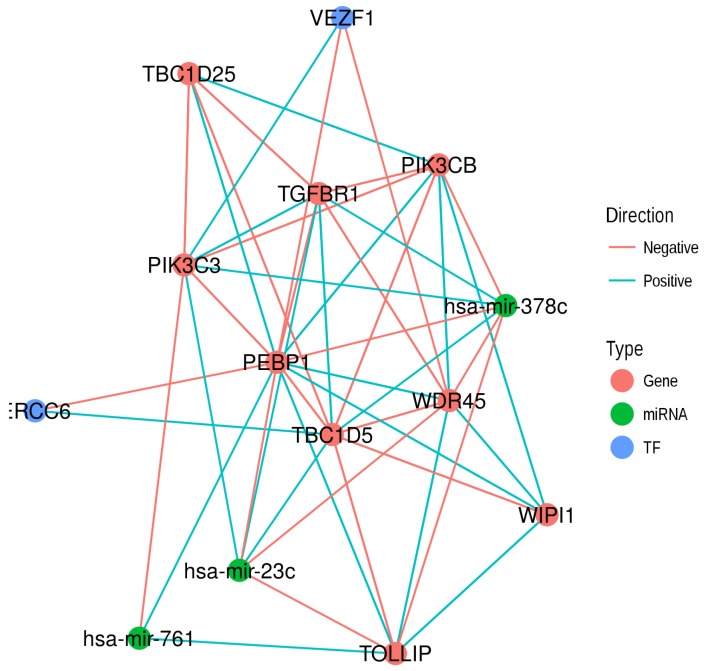
Network representation of gene interaction and regulation involving RKIP/PEBP1. A network graph shows nine gene products (*red*), three microRNAs (*green*) and two transcription factors (*blue*). Edges represent the expression correlation (negative, *red* and positive, *blue*) collected from different data sources. TF represents transcription factor.

To put it all together, we summarized the different gene interactions involving RKIP/PEBP1, EMT and autophagy gene products and their regulators as a graph (Figure 6). RKIP/PEBP1, eight interacting gene products, two transcription factors and three microRNAs are represented as nodes. Each pair of nodes was connected based on the evidence shown previously. Gene–gene interactions are the correlations of corresponding genes in different prostate sample types. Transcription factors and microRNAs were connected to their targets as identified by the common regulator analysis based on the cRegulome data.

### 2.7. Validation of Selected Gene Product Correlations with RKIP/PEBP1

**Figure 7:**
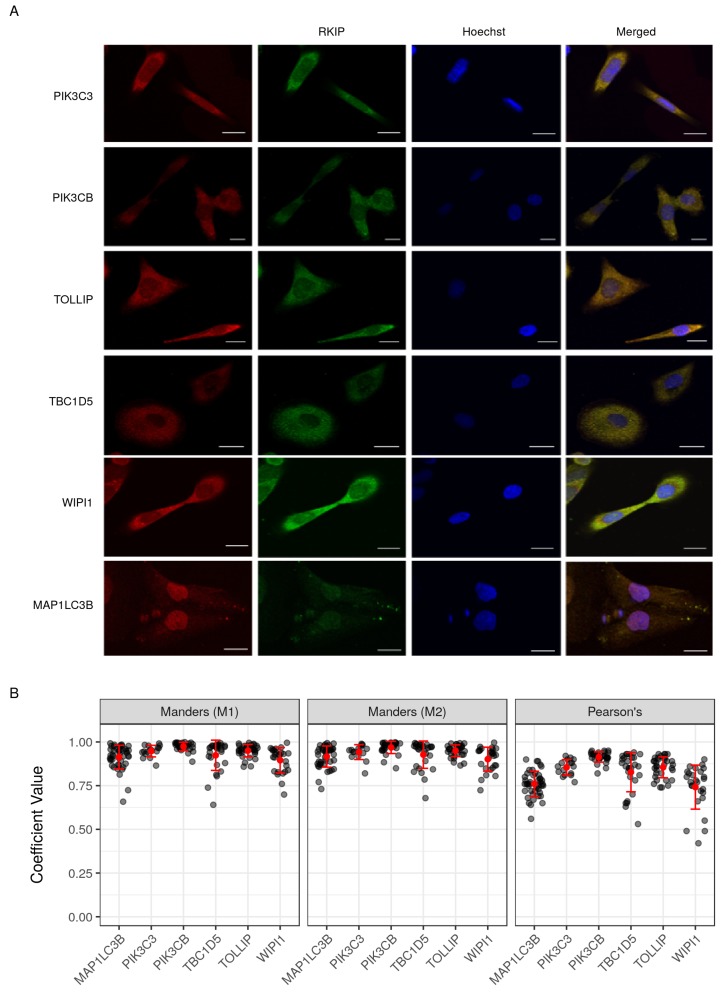
Co-localization of RKIP/PEBP1 with autophagy-related gene products. (**A**) immunohistochemistry. Co-localization images between RKIP/PEBP1 and autophagy gene products (PIK3C3, PIK3CB, TOLLIP, TBC1D5, WIPI1 or MAP1LC3B) in human prostate cancer cell line DU145 were obtained from the confocal Olympus FV-1000 microscope (Olympus Corporation, Tokyo, Japan). Nucleus was stained by Hoechst (300 ng/mL). Scale, 10 μm; (**B**) degree of co-localization between RKIP/PEBP1 and binding targets. The graphs (**left** two M1 and M2) represent the comparative mean Manders’ coefficient. Manders’ M1 and M2 values were taken above the auto-threshold of the green channel or red channel, respectively. The graph (**right**) shows the Pearson’s correlation coefficient of the co-localization targeted proteins. These values were calculated from variously selected regions of interest (*n* = 16 to 43).

To assess the novel interaction of RKIP/PEBP1 with autophagy and EMT gene products, five interactions were selected for the immunocytochemical analysis in the human prostate cancer cell line. DU145 cells were incubated with anti-rabbit RKIP antibody and one of PIK3C3, PIK3CB, TOLLIP, TBC1D5 or WIPI1 mouse antibodies. Co-localization of RKIP/PEBP1 and each of the five proteins was determined by the confocal fluorescence microscopy analysis (Figure 7A). In all five cases, RKIP/PEBP1 co-localized with its interacting partner as demonstrated by the high and significant (*p* < 0.01) Pearson’s and Manders’ coefficients (Figure 7B). In particular, both M1 and M2 Manders’ coefficients showed very similar patterns, indicating that the estimated amount of the co-localizing proteins from one channel was consistent with that from the other channel. Furthermore, all RKIP/PEBP1 co-localization estimates to the five proteins (PIK3C3, PIK3CB, TOLLIP, TBC1D5 or WIPI1) were similar to MAP1LC3B, which was shown previously to interact with RKIP/PEBP1 [3]. Finally, we verified three proteins (CTNNB1, PARK7 and NF1), which were previously reported as RKIP/PEBP1-interacting proteins through literature text-mining analysis. All three proteins co-localized very similarly with RKIP/PEBP1 (Supplementary Figure S3).

## 3. Discussion

Using the weighted-gene co-expression network analysis of a public-access gene expression dataset, we found that RKIP/PEBP1, an anti-tumor protein, potentially interacts with several key gene products in the autophagy and EMT gene sets during the development of prostate cancer. These gene products included TGFBR1, members of the WD Repeat Domain, PI3K and TBC families. We further showed that the co-expression network of these gene products was preserved in several independent datasets of prostate cancer. Finally, a selected group of the reported interaction was validated by in vitro co-localization assay in human prostate cancer cells.

The loss of RKIP/PEBP1 was initially connected to the development of prostate cancer and later to a few other cancer types [1]. In accordance with these findings, our analysis show that the expression of RKIP/PEBP1 was lower in metastatic tissues compared to benign and primary prostate tumor tissues. Moreover, RKIP/PEBP1 was part of a cluster of highly co-expressed gene products, the brown module, which was inversely correlated with the samples’ phenotypes (Figure 2A). Although a few of these potential interactions were reported previously, in this study, we further present several novel interactions that might explain the possible role of RKIP/PEBP1 in autophagy and EMT during the development of metastatic prostate cancer.

Several brown module members that correlated strongly with the expression of RKIP/PEBP1 have known functions in cancer development and progression (Table 3). Only one EMT gene product seems to be involved in this module; TGFBR1, which has a known polymorphism that is associated with cancer development [21]. The autophagy gene products included members of the WD repeat domain and TBC protein families, both of which were frequently mutated, associated or has reduced copy numbers in different types of cancers [15,16,19]. Similarly, two catalytic subunits of the PI3K complex, the catalytic subunit type 3 (PIK3C3) and the catalytic subunit beta (PIK3CB), contribute to cancer growth and metastasis [18,19]. The two catalytic subunits showed the novel interactions with RKIP/PEBP1. Understanding the interactions of RKIP/PEBP1 with these gene products may give insight into its role cancer development.

Interestingly, the two catalytic subunits of PI3K complex inversely contribute to the regulation of autophagy. PIK3CB activated by growth signals generally suppresses autophagy by blocking the ULK1/ULK2 activity via activation of mTORC1 signal [30]. By contrast, PIK3C3 binds to many autophagy regulators such as BECN1 or UVRAG and promotes induction of autophagy under certain conditions [31]. Previously, we also suggested that RKIP/PEBP1 negatively regulates autophagy [3]. In the current study, we observed that RKIP/PEBP1 had a negative expression correlation with PIK3C3 and conversely a positive expression correlation with PIK3CB as shown in Figure 4. These expression connections between RKIP/PEBP1 and PIK3C3 or PIK3CB were additionally observed in an independent analysis, which suggested that expression of RKIP/PEBP1 along with the two subunits was specifically regulated by a transcription factor (VEZF1) and three microRNAs (Figure 5). Overall, this co-expression network analysis supports our previous results in which RKIP/PEBP1 can inhibit autophagy by activation of mTORC1 via PIK3CB and by deterioration of LC3-lipidation via PIK3C3 signal.

Transforming growth factor beta 1 (TGFB1), secreted in the tumor micro-environment, modulates cancer growth through the specific binding to TGFBR1 and subsequent activation of intracellular signals [32]. The presence of TGFB1 increases the ability of cancer cells to metastasize by promoting invasion and migration [33,34]. In this study, we showed that TGFBR1 has a strong inverse correlation with RKIP/PEBP1 (Figure 4), suggesting that the elevated expression of RKIP/PEBP1 may decrease TGFBR1 and suppress cancer progression induced by TGFBR1-dependent signaling. According to an ongoing study from our laboratory, RKIP/PEBP1 might have some effects on TGFB1-induced EMT and cancer metastasis (data not published). Alternatively, TGFBR1 signaling may negatively modulate the intracellular level of RKIP/PEBP1 proteins via signaling intermediaries. This might be one way by which RKIP/PEBP1 keeps cancer cells in check, and the absence of it promotes EMT.

Multiple module members share a common regulator that contribute to cancer formation, with RKIP/PEBP1 (Table 4). Transcriptional regulators such as transcription factors and microRNAs control the expression level of coding genes under different circumstances. This provides a flexible mechanism for the cell to turn on/off certain genes in response to external stimuli or during development. Identifying common key regulators in one or more process can provide an insight into the particular role that they play in the cell physiology or the development of a disease. Two transcription factors—ERCC6 and VEZF1—simultaneously targeted RKIP/PEBP1 and one of TBC1D5 or PIK3C3 and WDR45, respectively. Both transcription factors were previously reported to be involved in tumorigenesis and chemoresistancy [22,24]. Three microRNAs, miR-378c, miR-761 and miR-23c, were low or moderately correlated with RKIP/PEBP1 and one or more of its interacting partners. Although most were not implicated directly in cancer, some of their variants were associated with cancer growth and invasion [26,27,28]. Even one of them, mir-23a was reported to induce the loss of RKIP/PEBP1 directly [29]. Interestingly, expression of RKIP/PEBP1 regulated by these microRNAs or transcription factors is consistently correlated to the expression level of two PI3K family proteins (PIK3C3 and PIK3CB) in the opposite direction of RKIP/PEBP1 in autophagy as shown previously [3]. The influence of upstream regulators may explain the observed strong correlations involving RKIP/PEBP1 in ways other than physical binding.

We used the WGCNA method to detect the conserved genetic networks of PEP, EMT and autophagy gene products that might contribute to the progression of prostate cancer [35,36]. Typically, the list of differentially expressed genes in three or more conditions are used as input to the WGCNA pipeline [4]. In this study, we limited the analysis to the probes that mapped uniquely to gene products in PEB, autophagy and EMT gene ontology terms. Limiting the analysis to a predefined set of genes means limiting the findings to the available annotations, losing signals from probes that are not part of the gene ontology terms and including probes that are not differentiated among the experimental conditions. However, this approach simplifies the steps of the analysis and the interpretation of the results. The detected modules would be biologically meaningful since they are made of nodes of known functions in the gene sets of interest. In addition, this approach allows including genes that might be highly correlated even if they are not strongly differentiated. Certainly, some of these genes are involved in cancer cell survival during the EMT and cancer metastasis by maintaining cancer homeostasis under several metabolic stress conditions.

## 4. Materials and Methods

### 4.1. Data and Annotation Sources

The gene ontology (GO) was used to identify the gene products of known functions in the terms phosphatidylethanolamine binding (PEB), epithelial to mesenchymal transition (EMT) and autophagy. PEB (GO:0008429) is a molecular function defined as interacting selectively and non-covalently with glycerophospholipids, where phosphatidyl group is transformed to hydroxyl group [37]. The term contains nine gene products including Phosphatidylethanolamine-binding protein 1 (PEBP1), Microtubule associated protein 1 light chain 3 (MAP1LC3), which are of particular interest to this study, and other gene products of similar functions. EMT (GO:0001837) and autophagy (GO:0006914) terms contains 36 and 108 gene products, respectively, and are defined as the cellular processes that allow cells to become migratory or digest parts of their own, in that order [30,38]. In total, 153 gene products were used through out the analysis to represent the three processes (Supplementary Table S1).

The main dataset in this analysis (GSE3325) was made of 13 microarrays from individuals with benign prostate tumor, primary and metastatic prostate cancer—4, 5 and 4 samples, respectively [7]. Total RNA from all samples was extracted and hybridized to the Affymetrix Human Genome U133 Plus 2.0 Array (GPL570). Here, we used the normalized probe intensities (expression matrix), accessible from the gene expression omnibus. In addition, a large set of human tissue samples of different types of prostate cancer, mainly adenocarcinmoas, were obtained from the NCI cancer genomic data server and used as a test set (Table 1).

### 4.2. Weighted-Gene Co-Expression Network Analysis

The weighted-gene co-expression network analysis (WGCNA) was applied on the gene expression dataset (GSE3325) of the human prostate tissues using an R package of the same name [39]. Briefly, a co-expression measure (Pearson’s correlation coefficient) was calculated between each pair of genes. The coefficients were raised to the power 5 to form an adjacency matrix. The adjacency matrix was then used to calculate the topological overlap similarity matrix (TOM). To detect clusters/modules and assign genes to them, a dissimilarity matrix is obtained (1-TOM) and used as distances between genes. A hierarchical clustering was then performed and a gene tree was built. Upon cutting the tree at a certain height, genes nearby were assigned to modules, referred to as colors (names are arbitrarily assigned). Eigengene vectors or the principal components (PC) were calculated from the expression of the respective members of each module and used as representative summaries. The significance of each module was assessed by its correlation with the samples’ phenotypes. Finally, module preservation analysis was performed by calculating various summary statistics on the detected modules in the test datasets [6].

### 4.3. Protein–Protein Interactions

The STRING database was used to identify the previously reported interactions of RKIP/PEBP1 with other PEB, EMT and autophagy gene products. In total, 153 gene symbols were mapped to the ENSEMBL IDs and used to construct the database query. The query output was matched against the WGCNA output to determine the potentially novel interactions (supplementary Table S3). The STRINGdb R package was used to map the IDs, construct and execute the query [40]. The STRING database provides reference and evidence for each of their reported interactions including but not limited to experimental reports, other databases and text-mining analysis.

### 4.4. Transcription Regulators Analysis

Transcription regulation analysis was performed using the cRegulome R package (preparing for publication). cRegulome obtains the transcription regulation data from two different resources: Cistrome Cancer for the transcription factors and the miRCancerdb for microRNAs [41,42]. The transcription factors targets are determined using ChIP-Seq date from different human tissues. The microRNAs targets are obtained from the TargetScan database. In both cases, the expression correlations of the regulators with their targets in various types of cancers are calculated using the cancer genome atlas RNA-Seq gene expression data.

### 4.5. Cell Culture and Immunocytochemistry

The DU145 human prostate cancer cells were seeded on cover glasses and cultured in DMEM containing 10% fetal bovine serum (FBS) at 37 ${}^{\circ }$C in 5% CO${}_{2}$ humidified atmosphere. At 60–80% confluence, cells were washed with phosphate buffered saline (PBS), fixed with 4% paraformaldehyde for 30 min, then permeabilized with 0.1% Triton X-100 in PBS for 60 min at room temperature. Finally, cells were incubated 2% bovine serum albumin (BSA) in PBS blocking solution for 60 min. For Immunostaining, each sample was simultaneously incubated with both of two primary antibodies (5–20 μg/mL each) including RKIP (polyclonal rabbit Ab, sc-28837) and one of the targeted gene monoclonal mouse antibodies (PIK3C3, sc-365404; PIK3CB, sc-376641; TOLLIP, sc-136152; TBC1D5, sc-376296; WIPI1, sc-100901; MAP1LC3B, sc-376404; NF1, sc-398267; CTNNB1, sc-7963, PARK7, sc55573 in 1% BSA in PBST (PBS + 0.1% Tween 20) at 4 ${}^{\circ }$C overnight. After incubation, cover glasses were washed twice in PBS and then incubated with both of two fluorescence-conjugated secondary antibodies (anti-mouse IgGκBP-CFL 594, sc-516178, and anti-rabbit IgG Alexa Fluor 488, A27034; 1:100 dilution factor in PBST + 1% BSA for 60 min at 37 ${}^{\circ }$C under dark. Nucleus was stained with Hoechst (300 ng/mL in 1% BSA in PBST for 10 min). After washing three times with PBST, cover glasses were embedded in $VectaMount^{TM}AQ$ mounting medium (Vector Laboratories, Inc., Burlingame, CA, USA) and covered with a coverslip. All images were obtained under the confocal microscope Olympus FV 1000 (Olympus Corporation, Tokyo, Japan).

### 4.6. Co-Localization Image Analysis

To analyze co-localization of RKIP/PEBP1 with proteins, we used the ImageJ software with coloc2 plug-in (Fiji) [43]. Multiple similar-sized symmetrical regions of interest (ROI) were selected on each dye. The background was subtracted from each ROI with a rolling ball radius at 50.0 pixels. The Pearson’s correlation and Manders’ split coefficients were calculated from ROIs (*n* = 16 to 43).

### 4.7. Software Environment and Reproducibility

The data were obtained, processed and analyzed in an R environment and using multiple Bioconductor packages [44,45]. The full analysis was done and reproduced in an isolated environment based on docker (bioconductor/release_base2) [46]. The scripts for reproducing the analysis, figures and tables are available at https://github.com/BCMSLab/rkip.

## 5. Conclusions

RKIP/PEBP1 interacts with EMT and autophagy-related gene products as part of the same functional unit in developing prostate cancer. Two distinct modules of highly co-expressed genes were identified. These modules were highly correlated with the progression of the disease of the prostate tissue. RKIP/PEBP1 showed novel gene–gene interactions with members of the EMT and autophagy gene sets, including TGFBR1, members of the WD Repeat Domain, PI3K and TBC families.

## Figures and Tables

**Table 1:** Studies of human prostate cancer subjects.

Study ID	Samples	Genes	Reference
prad.broad.2013	7	150	[8]
prad.broad	20	143	[9]
prad.fhcrc	171	149	[10]
prad.mskcc.cheny1.organoids.2014	10	148	[11]
prad.mskcc	150	151	[12]
prad.su2c.2015	118	152	[13]
prad.tcga.pub	333	152	[14]
prad.tcga	498	152	[14]

**Table 2:** Gene members in different modules/colors.

Module	Autophagy	Epithelial to Mesenchymal Transition	Phosphatidylethanolamine Binding
blue	*ABL1*, *ANXA7*, *ARSB*, *BNIP1*, *VPS51*, *CLTC*, *DAP*, *FOXO1*, *HMGB1*, *IFI16*, *NPC1*, *S100A8*, *S100A9*, *STK11*, *TMBIM6*, *TP53*, *UVRAG*, *SRPX*, *BECN1*, *USP10*, *ULK2*, *PLEKHM1*, *TECPR2*, *HDAC6*, *OPTN*, *RNF41*, *RGS19*, *ATG7*, *TM9SF1*, *WDR45*, *PARK7*, *VPS13A*, *VPS39*, *ULK3*, *PTPN22*, *TMEM208*, *NRBF2*, *RAB39A*, *FNBP1L*, *WIPI1*, *MAP1S*, *DRAM1*, *SUPT20H*, *VPS11*, *TIGAR*, *VPS18*, *PHF23*, *MAP1LC3B*, *VMP1*, *C19orf12*, *ATG10*, *EVA1A*, *WDR24*, *ATG4C*, *TRIM5*, *LRSAM1*, *RAB39B*, *LRRK2*, *DRAM2*, *SMCR8*	*BMP2*, *BMP7*, *FGFR2*, *FOXF2*, *HNRNPAB*, *RBPJ*, *LOXL2*, *S100A4*, *SNAI2*, *TGFB1*, *TGFB2*, *TGFBR3*, *WNT5A*, *DLG5*, *NOG*, *DDX17*, *LEF1*, *EPB41L5*, *FAM83D*, *LOXL3*, *RFLNB*	*ANXA11*, *MFGE8*, *PLTP*, *PEMT*, *CD300A*, *MAP1LC3A*
brown	*CTSD*, *RAB8A*, *TBC1D25*, *PIK3C3*, *PIK3CB*, *RAB1A*, *VCP*, *TFEB*, *ULK1*, *SQSTM1*, *HAP1*, *ATG5*, *NAPSA*, *RUBCN*, *TBC1D5*, *SIRT2*, *ATG4B*, *TECPR1*, *CHMP2B*, *VPS41*, *TRIM17*, *TOLLIP*, *ZKSCAN3*, *CHMP4B*, *RAB12*, *C9orf72*	*AMELX*, *CTNNB1*, *HGF*, *HIF1A*, *SNAI1*, *SOX9*, *TGFBR1*, *HMGA2*	*NF1*, *PEBP1*, *ESYT2*
yellow	*ITGB4*, *PGC*, *USP13*, *TMEM59*, *RB1CC1*, *GABARAPL2*, *CLEC16A*, *UBQLN2*, *SH3GLB1*, *WDR41*, *VTI1A*	*GSK3B*, *NOTCH1*, *WNT11*, *CUL7*, *WNT4*	

**Table 3:** Summary of RKIP/PEBP1 interactions.

Family	Protein	Name	Main Function
WD Repeat Domain	WDR45	WD Repeat Domain 45	Frequently mutated in lung adenocarcinomas [15].
	WIPI1	WD Repeat Domain, Phosphoinositide Interacting 1	High expression is associated with survival in hepatocellular carcinoma patients [16].
PI3K	PIK3C3	Phosphatidylinositol 3-Kinase Catalytic Subunit Type 3	Promote cancer growth through p62 [17].
	PIK3CB	Phosphatidylinositol-4, 5-Bisphosphate 3-Kinase Catalytic Subunit Beta	Mediates cancer metastasis [18].
TBC	TBC1D5	TBC1 Domain Family Member 5	Reduced copy number in breast cancer [19].
	TBC1D25	TBC1 Domain Family Member 25	
Other	TOLLIP	Toll Interacting Protein	Hypermethylated in response to sex hormones in prostate cancer cells [20].
	TGFBR1	Transforming Growth Factor Beta Receptor 1	Multiple polymorphisms are associated with cancer development [21].

**Table 4:** Common transcription factors of PEBP1 and interacting genes.

Factor	Name	Function
ERCC6	ERCC Excision Repair 6, Chromatin Remodeling Factor	A DNA-binding protein that is important in transcription-coupled excision repair. Several polymorphisms the gene coding region were associated with susceptibility to development of cancer and chemoresistancy [22,23].
VEZF1	Vascular Endothelial Zinc Finger 1	A transcriptional regulatory protein that is involved in angiogenesis. Contribute to the epigenetic aberrations and the associated tumorigenesis [24,25].
hsa-miR-378c	Close relative (hsa-miR-378a)	Inhibits cell growth and enhances apoptosis in cancer [26].
hsa-miR-761		Enhances cancer growth, migration and invasion [27].
hsa-miR-23c	Close relative (hsa-miR-23a)	Associated with autophagy, loss of RKIP/PEBP1 and multiple tumors [28,29].

## Supplementary Materials

The following are available online at http://www.mdpi.com/2072-6694/10/8/273/s1, Figure S1. Scale free topology for multiple power values. Figure S2. Module preservation ranks across multiple prostate cancer datasets. Figure S3. Co-localization of RKIP with previously reported interacting proteins. Table S1. Gene members of the three gene ontology terms. Table S2. PEB interactions with autophagy and EMT. Table S3. Summary of previously reported and novel PEBP1 interactions.

## Abbreviations

RKIP	Raf Kinase Inhibitor Protein
PEBP1	Phosphatidylethanolamine Binding Protein 1
PEB	Phosphatidylethanolamine Binding
EMT	Epithelial to Mesenchymal Transition
WGCNA	Weighted-Gene Co-expression Network Analysis
GO	Gene Ontology
TOM	Topological Overlap Matrix
PC	Principal Component
ROI	Region of Interest
RNA-Seq	RNA Sequencing
ChIP-Seq	Chromatin Immunopreciptation followed by DNA Sequencing
NCI	National Cancer Institute

## Appendix A. A Note on Reproducing the Analysis

This is a detailed description of the details required to reproduce the analysis from the source code. We first introduce the way to obtain the source code, the software environment and the commands to run the analysis script and generate the figures and tables that appear in this manuscript.

### Appendix A.1. Setting up the Docker Environment

The analysis was run on a docker image based on the the latest bioconductor/release_base2. Other R packages were added to the image and were made available as an image that can be obtained and launched on any local machine running docker.

$ docker pull bcmslab/rkip
$ docker run -it bcmslab/rkip bash

### Appendix A.2. Obtaining the Source Code

The source code is hosted publicly on a repository on github in a form of research compendium. This includes the functions used throughout the analysis as an R package, the scripts to run the analysis and finally the scripts to reproduce the figures and tables in this manuscript. From within the container, git can be used to cloned the source code. The cloned repository contains a sub-folder called ’analysis/scripts/’ which can be used to reproduce the analysis from scratch

- ’get_data.R’ This script downloads several datasets from different sources in preparation of the analysis
- ’analysis.R’ This script loads the required libraries, downloads the data and runs all the steps of the analysis described in the manuscript
- ’figures/’ A sub-folder with a separate file for each graph in the manuscript.
- ’tables/’ A sub-folder with a separate file for each table in the manuscript.

The following code clones the repository containing the source code.

$ git clone https://github.com/BCMSLab/rkip

### Appendix A.3. Running the Analysis

The analysis scripts is organized to be run using a single ’make’ command. This will first load the necessary functions and run the main analysis and save the data in an R object ’data/rkip_wgcna.rda’. This will be used to generate the figures and graphs. In addition, a log file is generated in the sub-folder ’log/’ for each script, which can be used for troubleshooting.

To do that, the ’make’ command should be invoked from withing the ’analysis/’ sub-folder.

$ **cd** rkip/analysis/
$ make

### Appendix A.4. Details of the R Environment

The version of R that was used to perform this analysis is the 3.4.3 (2017-11-30) on x86_64-pc-linux-gnu. The ’DESCRIPTION’ file in the main repository contains further details about the dependencies and the license of this work.
