# Supplementary material for: Functional Linkage of RKIP to the Epithelial to Mesenchymal Transition and Autophagy during the Development of Prostate Cancer
Source: Cancers (Basel). 2018 Aug 16;10(8):273. doi: 10.3390/cancers10080273 (PMC6115972; doi:10.3390/cancers10080273)
Supplement: Supplementary file 1 [file cancers-10-00273-s001.pdf]

Table S1: Gene products in the three gene ontology terms.

Term	Gene Products
autophagy (GO:0006914)	*ABL1*, *ANXA7*, *ARSB*, *BNIP1*, *VPS51*, *CLTC*, *CTSD*, *DAP*, *FOXO1*, *HMGB1*, *IFI16*, *ITGB4*, *RAB8A*, *NPC1*, *TBC1D25*, *PGA5*, *PGC*, *PIK3C3*, *PIK3CB*, *RAB1A*, *S100A8*, *S100A9*, *STK11*, *TMBIM6*, *TP53*, *UVRAG*, *VCP*, *XBP1*, *TFEB*, *SRPX*, *ULK1*, *BECN1*, *SQSTM1*, *USP13*, *HAP1*, *USP10*, *ATG5*, *NAPSA*, *TMEM59*, *ULK2*, *RUBCN*, *TBC1D5*, *RB1CC1*, *PLEKHM1*, *TECPR2*, *HDAC6*, *OPTN*, *RNF41*, *RGS19*, *ATG7*, *TM9SF1*, *WDR45*, *PARK7*, *GABARAPL2*, *SIRT2*, *ATG4B*, *VPS13A*, *CLEC16A*, *VPS39*, *TECPR1*, *CHMP2B*, *ULK3*, *PTPN22*, *VPS41*, *TMEM208*, *UBQLN2*, *NRBF2*, *SH3GLB1*, *TRIM17*, *RAB24*, *TOLLIP*, *RAB39A*, *FNBP1L*, *WIPI1*, *MAP1S*, *WDR41*, *DRAM1*, *SUPT20H*, *VPS11*, *UBQLN4*, *TIGAR*, *VPS18*, *PHF23*, *TBC1D17*, *ZKSCAN3*, *MAP1LC3B*, *VMP1*, *RAB1B*, *C19orf12*, *ATG10*, *EVA1A*, *WDR24*, *ATG4C*, *TRIM5*, *LRSAM1*, *RNF185*, *RAB39B*, *LRRK2*, *DRAM2*, *CHMP4B*, *SMCR8*, *VTI1A*, *RAB12*, *C9orf72*, *NHLRC1*, *BECN2*, *PGA3*, *PGA4*
epithelial to mesenchymal transition (GO:0001837)	*AMELX*, *BMP2*, *BMP7*, *CTNNB1*, *DDX5*, *FGFR2*, *FOXF2*, *GSK3B*, *HGF*, *HIF1A*, *HNRNPAB*, *RBPJ*, *LOXL2*, *NOTCH1*, *S100A4*, *SNAI2*, *SNAI1*, *SOX9*, *TGFB1*, *TGFB2*, *TGFBR1*, *TGFBR3*, *WNT5A*, *WNT11*, *HMGA2*, *DLG5*, *NOG*, *CUL7*, *TRIM28*, *DDX17*, *LEF1*, *WNT4*, *EPB41L5*, *FAM83D*, *LOXL3*, *RFLNB*
phosphatidylethanolamine binding (GO:0008429)	*ANXA11*, *MFGE8*, *NF1*, *PEBP1*, *PLTP*, *PEMT*, *CD300A*, *ESYT2*, *MAP1LC3A*

Table S2: PEB interactions with autophagy and EMT.

Module	PEB	Autophagy	EMT
blue	*ANXA11*	*ANXA7*, *ATG4C*, *CLTC*, *FOXO1*, *HDAC6*, *IFI16*, *MAP1LC3B*, *MAP1S*, *PARK7*, *PHF23*, *RAB39A*, *RNF41*, *S100A9*, *SMCR8*, *SRPX*, *SUPT20H*, *TMEM208*, *TP53*, *VPS11*, *VPS18*, *VPS39*, *WDR45*, *WIPI1*	*BMP7*, *DDX17*, *DLG5*, *EPB41L5*, *FAM83D*, *HNRNPAB*, *LOXL2*, *LOXL3*, *SNAI2*, *TGFB1*, *WNT5A*
	CD300A	*HMGB1*, *PARK7*, *RGS19*	*DLG5*
	ESYT2	*PTPN22*, *STK11*, *TM9SF1*	
	MAP1LC3A	*MAP1S*, *NRBF2*, *PARK7*, *RAB39A*, *RNF41*, *SMCR8*, *SUPT20H*, *TIGAR*, *TMEM208*, *TRIM5*, *ULK2*, *VPS39*	*SNAI2*, *TGFB1*, *TGFB2*
	MFGE8	*RAB39B*, *WDR24*	*NOG*, *TGFB2*
	NF1	*NRBF2*	
	PEBP1	*WDR45*, *WIPI1*	
	PEMT	*TMBIM6*	
	PLTP	*RNF41*, *SRPX*, *TRIM5*, *VPS39*	*S100A4*, *SNAI2*, *TGFB1*
brown	*ESYT2*	*TECPR1*	*TGFBR1*
	MAP1LC3A	*NAPSA*, *PIK3C3*, *RAB1A*, *RAB8A*, *SIRT2*, *TFEB*, *ULK1*	
	MFGE8	*RAB12*	
	NF1	*PIK3C3*, *RAB1A*, *TBC1D5*, *ZKSCAN3*	
	PEBP1	*PIK3C3*, *PIK3CB*, *TBC1D25*, *TBC1D5*, *TOLLIP*	*TGFBR1*
	PLTP	*RAB8A*, *RUBCN*, *SIRT2*, *TRIM17*, *ULK1*	
yellow	*ANXA11*	*GABARAPL2*	*GSK3B*, *WNT11*
	PLTP		*WNT11*

Table S3: Summary of previously reported and novel RKIP/PEBP1 interactions.

Category	Evidence	Gene
Autophagy	coexpression	PARK7
	novel	PIK3C3, PIK3CB, TBC1D25, TBC1D5, TOLLIP, WDR45, WIPI1
	textmining	TP53, PARK7, HMGB1
EMT	novel	TGFBR1
	textmining	CTNNB1, HMGA2
PEB	experiments	NF1
	textmining	NF1

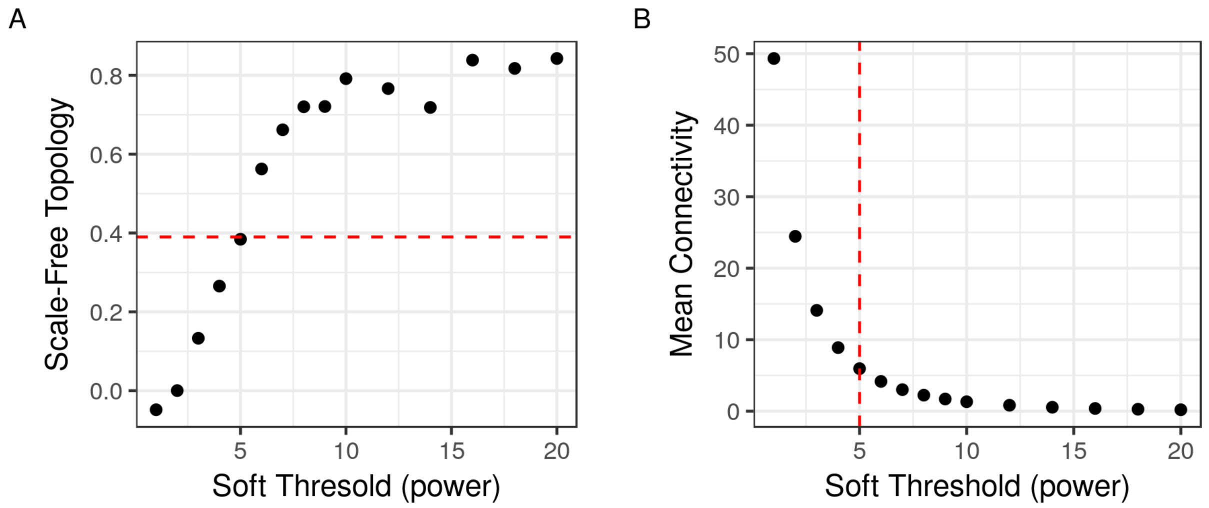

Figure S1: Scale free topology for multiple power values. For each power value, a scale free topology index was calculated. (**A**) The fit indices, the slopes multiplied by the R squared (R^2) values, are shown as points for each power value. (**B**) The mean connectivity, average edges shared by a node, for the resultant network at each power value are shown as points. Red lines represent the choice of power that satisfies both high R^2 values and high connectivity.

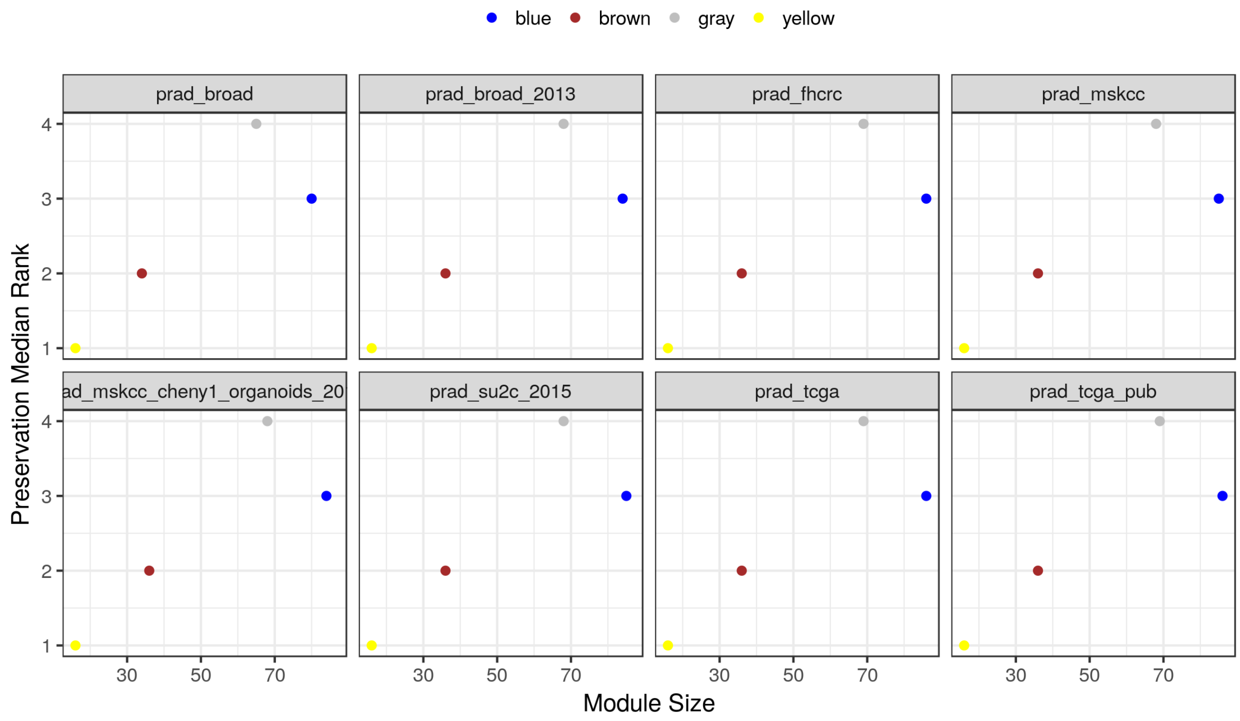

Figure S2: Module preservation ranks across multiple prostate cancer datasets. The GSE3325 dataset was used to detect the highly co-expressed modules among PEB, EMT and autophagy genes (87, *blue*; 37, *brown*; 18, *yellow*; and gray, randomly assigned). The detected modules were used as a reference to calculate several preservation statistics in eight independent datasets of prostate cancer. The median ranks of the preservation statistics and the sizes of four modules (blue, brown and yellow; gray, randomly assigned) are shown as colored points.

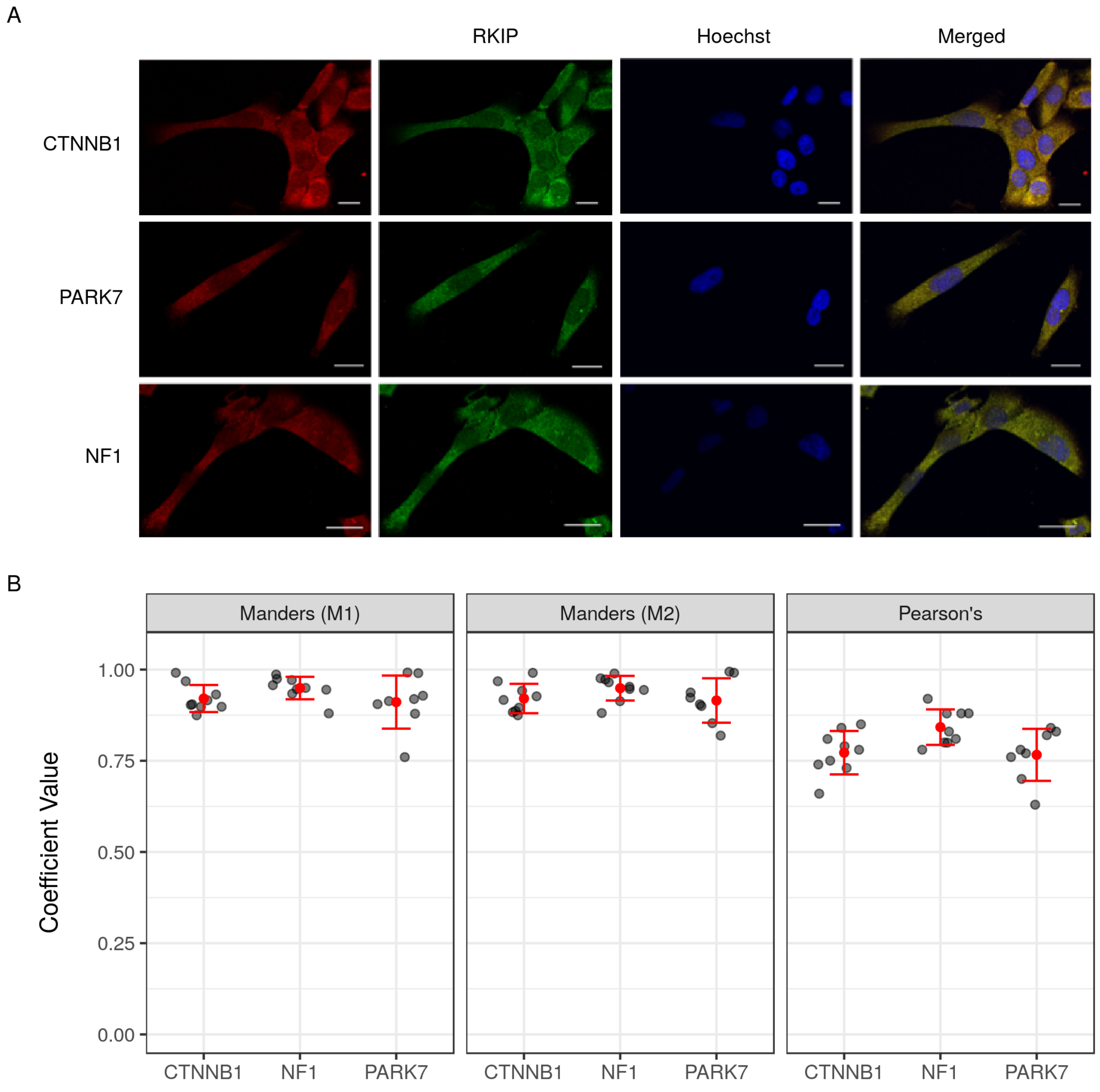

Figure S3: Co-localization of RKIP/PEBP1 with previously reported interacting proteins. (**A**) Immunohistochemistry. Co-localization images between RKIP/PEBP1 and autophagy gene products (CTNNB1, PARK7 and NF1) in human prostate cancer cell line DU145 were obtained from the confocal Olympus FV-1000 microscope (OLYMPUS CORPORATION, Japan). Scale, 10 μm. Nucleus was stained by Hoechst. (**B**) Degree of co-localization between RKIP/PEBP1 and binding targets. The graphs (*left* two M1 and M2) represent the comparative mean Manders' coefficient. Manders' M1 and M2 values were taken above the auto-threshold of green or red channel, respectively. The graph (*right*) shows the Pearson's correlation coefficient of the co-localization targeted proteins. These values were calculated from variously selected ROIs (n=5 to 10).
